# What Should be Prepared for the Mutual Accreditation for Medical Health Licenses When Korea Opens to Physicians and Dentists from the Rest of the World

**DOI:** 10.3352/jeehp.2007.4.2

**Published:** 2007-05-14

**Authors:** Sun Huh

**Affiliations:** Department of Parasitology, College of Medicine and Institute of Medical Educations, Hallym University, Chuncheon, Korea.

The "Law of the Designation and Operation of the Free Economic Zone", which goes into effect September 28, 2007, will allow foreigners to establish medical institutes in the Free Economic Zone, which includes the Korean cities of Busan-Jinhae, Incheon, and Kwangyang. Physicians and dentists with a license from a foreign country will be allowed to practice in those institutes, and treat Korean patients. The government anticipates that this policy will reduce the expenses of medical travel, improve service for Koreans, and strengthen the competitiveness of Korean medical institutes in the global market. The policy's primary objective is to aid foreigners in Korea, but it is also designed to increase foreign investment in Korea. The purpose of these policies is very clear. However, regarding the basic health rights of people in Korea, both Koreans and foreigners, the accreditation of foreign licenses, without the stipulation that such licenses conform to Korean law, does not meet global standards. If the Korean Government wants to allow foreign medical health personnel to practice in Korea, a mutual agreement on the accreditation of licenses between corresponding countries is necessary. A medical VISA is not an unconditional accreditation, and test-equating procedures are required at the levels of undergraduate training and graduate training, as well as that of continuing medical education.

Also on July 6, 2006, the Minister of Health and Welfare of the Republic of Korea announced the revision of its "Enforcement Regulation for Medical Law," which was rejected by the Minister of Government Legislation since it violated the "Medical Law." The proposed revised content follows:

Foreigners with a medical health license, such as physicians, dentists, or nurses, can treat people from their homeland who are residing in Korea, when employed by a general hospital or clinic as defined under "Medical Law," to guarantee the health rights of foreigners residing in Korea.

Because the Korean Government is greatly concerned about the health of foreigners working in Korea, this kind of revision was launched. However, because the revision targets temporary immigrant laborers, the medical expenses will be not be great. The general hospitals or clinics should decide on whether to hire foreign medical health personnel after considering the number of patients it commonly treats. Although the government will allows medical institutes to hire foreigners, active recruitment depends on the market. It is questionable whether other Organization for Economic Co-operation and Development (OECD) countries offer this kind of system to foreigners residing in their countries. Although the revision was rejected by the Minister of Government Legislation, it was said that officer of Health and Welfare will try again the revision of "Medical Law" instead of "Enforcement Regulation for Medical Law." If it is tried, there is a high possibility that this revision is passed.

Furthermore, Korea and the United States announced the Korea-United States Free Trade Agreement (FTA) on April 2, 2007. A "specialist service working group" will be created to discuss the mutual accreditation of specialists. The first round will include the fields of engineering, architecture, and veterinary medicine. It is uncertain whether medical health personnel will be included in the next round. Korean Medical Association (KMA), the official organization of physicians in Korea, asked the Korean delegates of the FTA for a mutual medical VISA quota to the United States, but this request was not considered at first. Corresponding officers of governments have noted that they would try to negotiate with Congress of the United States since it is the area of immigration. Korea also began discussing the FTA with Canada on July 2005, and talks with the European Union will begin on May 7, 2007.

However, before proposing mutual accreditation for medical health licenses, it may first be necessary to develop a scientific foundation for test equating and educational standardization. The mutual agreement of medical health licenses may become an ongoing issue. Before any such agreement can be made, or a one-sided inclusion of foreigners is accepted, test equating should be considered and the data should serve as a scientific base. In the very near future, Korea will be open to foreign medical health personnel. Some foreigners will have the opportunity to practice in Korea. It is time to consider the health rights of the people living in Korea, and to question why each country has its own accreditation system. It may be difficult to equate licensing examinations between corresponding countries because these are high-stake tests. Due to different cultural backgrounds, and different epidemiological data, it is also difficult to test similar items in such exams. In this case, common items can be prepared in each language, but such an approach will not meet the strict definition of test equating until after they have been tested for validity and reliability. For this kind of test equating, at each level of education, internet- and computer-based testing (iB-CBT) are the best tools. Computerized adaptive testing (CAT) can also be used. This kind of test equating can also be applied to clinical skill tests or to objective structured clinical examinations (OSCE) via iB-CBT. To assess such test equating, it should be applied to at least two medical schools in two different countries or, ideally, to several medical schools in several countries. Then test equating between hospitals and between continuing medical education institutes could be launched. Test equating may be one of methods of the evaluation of medical health personnel of different countries. To this end, psychometrician's consultation is not only necessary, but physicians must understand psychometrics.

